# Factors Influencing Anxiety Among WeChat Users During the Early Stages of the COVID-19 Pandemic in Mainland China: Cross-sectional Survey Study

**DOI:** 10.2196/24412

**Published:** 2021-05-17

**Authors:** Changqing Zou, Weiyu Zhang, Kristin Sznajder, Fengzhi Yang, Yajing Jia, Ruqing Ma, Can Cui, Xiaoshi Yang

**Affiliations:** 1 Department of Humanities and Social Sciences China Medical University Shenyang China; 2 Department of Social Medicine, School of Public Health China Medical University Shenyang China; 3 Department of Public Health, College of Medicine Pennsylvania State University Philadelphia, PA United States

**Keywords:** anxiety, COVID-19, information seeking behavior, positive psychological response, health information, public health emergency, mental health, online survey, China, cross-sectional study

## Abstract

**Background:**

The rapid outbreak of COVID-19 around the world has adversely affected the mental health of the public. The prevalence of anxiety among the public has increased dramatically during the COVID-19 pandemic. However, there are few studies evaluating the effects of positive psychological responses and information-seeking behaviors on anxiety experienced among social media users during the COVID-19 pandemic.

**Objective:**

This study evaluated the prevalence of anxiety and its associated factors among WeChat users in mainland China during the early stages of the COVID-19 pandemic.

**Methods:**

From February 10 to February 24, 2020, a nationwide, web-based cross-sectional survey study was carried out using convenience sampling. Participants’ levels of anxiety, positive psychological responses, and information-seeking behaviors were assessed. The survey was distributed among WeChat users via the WeChat smartphone platform. Chi-square tests and multivariable logistic regression analyses were performed to examine the factors associated with anxiety.

**Results:**

This study found that the prevalence of anxiety (Generalized Anxiety Disorder 7-item [GAD-7] scale score ≥7) among WeChat users in China was 17.96% (446/2483) during the early stages of the COVID-19 pandemic. Results of multivariable logistic regression analysis showed that information-seeking behaviors such as cannot stop searching for information on COVID-19, being concerned about the COVID-19 pandemic, and spending more than 1 hour per day consuming information about the pandemic were found to be associated with increased levels of anxiety. Additionally, participants who chose social media and commercial media as the primary sources to obtain information about the COVID-19 pandemic were found more likely to report anxiety. Conversely, participants who were confident or rational about the COVID-19 pandemic were less likely to report anxiety.

**Conclusions:**

This study found that positive psychological responses and information-seeking behaviors were closely associated with anxiety among WeChat users during the COVID-19 pandemic in China. It might be paramount to enhance mental well-being by helping people respond to the COVID-19 pandemic more rationally and positively in order to decrease symptoms of anxiety.

## Introduction

In January 2020, the World Health Organization classified COVID-19 as a public health emergency of international concern [1,2]. COVID-19 spread rapidly to 208 countries and regions and became a global pandemic, resulting in more than 30 million cases of infection and over 950,000 deaths worldwide, as of September 18, 2020 [3,4]. The sudden outbreak of COVID-19 adversely impacted the daily life and mental health of the public, which demanded urgent solutions [5-12].

The COVID-19 pandemic generated a psychological crisis among the public as the prevalence of mental disorders, including anxiety and depression, increased [13-18]. Various sources of information, including official media channels, social media, and the local government or community, could significantly affect the public’s psychological responses to emergency events and their mental well-being. Recent research has found that excessive information-seeking behaviors have been closely related with mental disorders, including anxiety and depression, during the COVID-19 pandemic [19,20]. Individuals with anxiety tend to focus on negative information and correspondingly experience negative psychological responses [21]. The frequency and daily duration of social media use was higher in areas with a higher number of COVID-19 cases. High levels of social media consumption were associated with increased mental distress, especially among individuals with high levels of fear [22]. The metacognitive model illustrates that certain behaviors such as controlling behaviors, reassurance seeking, and checking behaviors are positively associated with anxiety [23-25]. Thus, excessive media consumption could lead to harmful psychological responses, which could increase anxiety among these individuals during the COVID-19 pandemic.

Positive psychological responses to the COVID-19 pandemic among the public play a crucial part in reducing anxiety. Positive thoughts and attitudes could help individuals cope with stressors [26]. A recent study found that hope can contribute to anxiety prevention [27]. The theory of rational emotive behavior therapy indicates that rational beliefs can alleviate symptoms of anxiety and other mental distress [28,29]. Additionally, cognitive behavioral models of health anxiety indicate that negative emotions and misinterpretations of health-related stimuli could increase the chances of developing anxiety [30-33]. Therefore, promoting positive psychological responses and initiating emotion regulation and positive perceptions of health-related information, such as keeping a positive attitude and being rational, are necessary for the public to be able to better cope with stress [34].

Researchers have studied the pathogenic and epidemiological characteristics, epidemiological trends, as well as the prevention and treatment of COVID-19 [35-43]. However, studies focused on the relationship between information-seeking behaviors and anxiety during the COVID-19 pandemic are sparse. Therefore, this study focused on the evaluation of the prevalence of anxiety and its association with positive psychological responses and information-seeking behaviors among users of WeChat—the most popular social media platform in Mainland China—during the COVID-19 pandemic.

## Methods

### Study Participants and Procedure

In order to examine the prevalence of anxiety among WeChat users during the COVID-19 pandemic, a cross-sectional study design using convenience sampling was employed via a web-based WeChat platform developed by the Department of Environmental Health of China Medical University in China. Due to the urgency of the COVID-19 pandemic and the necessity of timely acquisition, the questionnaire was first uploaded to this web-based WeChat platform. Next, the survey was distributed via all the open WeChat groups of the research assistants, to render the survey easily available to their close contacts. The survey was distributed between February 10 and February 24, 2020, during which we achieved a sample size that was adequate in accordance with previous related studies [44-47]. The questionnaire comprised the validated Chinese version of the Generalized Anxiety Disorder 7-item (GAD-7) scale, questions on positive psychological responses, and questions on information-seeking behaviors practiced during the early stage of the COVID-19 pandemic. The questionnaire took about 20 minutes to complete.

The inclusion criteria for participation in this study were as follows: at least 18 years of age, ability to read and write Chinese, ability to complete the web-based questionnaire by themselves using a smartphone, ability to offer electronic signed informed consent, and voluntary participation. The exclusion criteria were as follows: undergoing any therapy for psychological illness, history of any drug dependence, and diagnoses of any diseases or impairments that would prevent them from completing the questionnaire independently. The questionnaire had been preset for submission only after all the questions were answered within the range of the selected choices. Only data from complete questionnaires were analyzed.

### Ethics Statement

This study was carried out in conformance with the Declaration of Helsinki (1989). The study protocols were approved by the Ethics Committee of China Medical University.

### Demographic Characteristics of WeChat Users

The survey collected participants’ demographic characteristics, including gender (male or female), age, marital status (married or other), occupation, education, and monthly income. Occupation was further classified into government worker, health care worker, professional staff (teacher, lawyer, journalist, etc), employee of enterprises, commercial personnel, soldier, student, and other. Education was further classified into junior college or below, bachelor’s degree, and master’s degree or above. Monthly income was categorized as follows: ≤¥5000 (≤US $725.19), ¥5001-10,000 (US $725.34-1450.39), and >¥10,000 (>US $1450.39).

### Measurement of Anxiety

The validated Chinese version of the GAD-7, one of the most reliable tools to measure generalized anxiety disorder, was used to assess anxiety among the study participants during the early stages of the COVID-19 pandemic [48]. Psychological problems related to anxiety were evaluated with a 4-point Likert-type scale, with options including “not at all sure=0”, “several days=1”, “over the half of the days=2” and “nearly every day=3,” resulting in a total score ranging from 0 to 21. The cutoff score for anxiety was set at ≥7, based on the total GAD-7 score [49-51], with Cronbach α=.939 for GAD-7. The results of the factor analysis indicated that the seven items of the positive psychological responses tested were extracted as one component, which contributed to 73.518% of the variance (Kaiser-Meyer-Olkin [KMO]=0.935, *P*<.001]. The indicators of reliability and validity were good.

### Measurement of Positive Psychological Responses

Positive psychological responses included “being confident,” “being hopeful,” and “being rational” in the past 2 weeks. They were measured by “yes” or “no” questions (Cronbach α=.788). The results of the factor analysis indicated that the three items of the positive psychological responses tested were extracted as one component, which contributed to 70.932% of the variance (KMO=.638, *P*<.001). The indicators of reliability and validity were good.

### Measurement of Information-Seeking Behaviors

Information-seeking behaviors were assessed using three self-developed questions; these behaviors were assessed in the following four categories: (1) cannot stop searching for information about the COVID-19 pandemic, (2) being concerned about the COVID-19 pandemic, (3) time spent consuming information about the COVID-19 pandemic (ie, <1 h, 1-2 h, or ≥3 h), and (4) sources of information about the COVID-19 pandemic (social media and commercial media, central and local official media, basic-level government media, and community media). Responses to the item “cannot stop searching for information about the COVID-19 pandemic” included “agree,” “not sure,” and “disagree.” Whether participants were concerned about the COVID-19 pandemic and their sources of information about the pandemic were assessed by “yes” or “no” questions.

### Statistical Analyses

SPSS software (version 23.0; IBM Corp) was used to perform all statistical analyses. Chi-square tests were applied to assess bivariate associations with anxiety and the variables of interest. Anxiety was assessed using the binary variable (*anxiety* or *no anxiety*, as measured by the GAD-7 scale). Multivariable logistic regression analysis showed that the factors associated with anxiety were assessed while controlling for confounding variables. The variables included in the multivariable logistic regression analysis included age as a continuous variable and other variables as categorized variables. The responses were not included in the analysis when more than 95% of individuals had the same response to the categorical independent variables. Findings were considered statistically significant when a two-tailed *P* value was <.05.

## Results

### Demographic Characteristics and Prevalence of Anxiety Among WeChat Users

In this study, we observed that 446 of 2483 (17.96%) Chinese WeChat users who participated in our survey experienced anxiety. A total of 2501 adults participated in this survey and 2483 of them provided complete and logical answers, resulting in a valid response rate of 99.28%. The demographic characteristics of the participants and results from the bivariate analysis of anxiety are presented in Table 1. The mean age of participants was 34 (SD 12.82) years. Of the 2483 participants, 1550 (62.42%) were female, and 278 (17.94%) of them reported having experienced anxiety. About half of the participants (1239/2483, 49.90%) were married. Their occupations were classified into three groups according to their distribution and frequency: (1) government worker, health care worker, teacher, lawyer, or journalist (515/2483, 20.74%); (2) student (954/2483, 38.42%); and (3) other, including employee of enterprises, commercial personnel, or soldier (1014/2483, 40.84%). More than half of the participants had a bachelor’s degree (1394/2483, 56.14%), and many had a master’s degree or above (800/2483, 32.22%). The remaining 11.64% (289/2483) participants whose educational level was “some college or below” showed significantly higher prevalence of anxiety (61/289, 21.11%) than the other two groups. With regard to the distribution of monthly income, 40.03% (994/2483) of the participants earned ≤¥5000 (≤US $725.19), 35.60% (884/2483) earned ¥5001-10,000 (US $725.34-1450.39), and 24.37% (605/2483) earned >¥10,000 (>US $1450.39).

**Table 1:** Prevalence of anxiety and associated factors among study participants (N=2483).

Variable			Participants, n (%)	Anxiety experienced, n (%)	No anxiety experienced, n (%)	Chi-square (*df*)		*P* value
**Demographic characteristics**								
	**Gender**					0.004 (1)		.96
		Male	933 (37.60)	167 (17.90)	766 (82.10)			
		Female	1550 (62.40)	278 (18.00)	1272 (82.00)			
	**Age (years)**					1.985 (1)		.17
		≤35	1474 (59.33)	278 (18.86)	1196 (81.14)			
		>35	1009 (40.67)	168 (16.65)	841 (83.35)			
	**Marital status**					0.071 (1)		.79
		Married	1239 (49.90)	220 (17.76)	1019 (82.24)			
		Other	1244 (50.10)	226 (18.17)	1018 (81.83)			
	**Occupation**					4.042 (2)		.13
		Government worker, health care worker, teacher, lawyer, journalist	515 (20.74)	85 (16.50)	430 (83.50)			
		Student	954 (38.42)	160 (16.77)	794 (83.23)			
		Other	1014 (40.84)	201 (19.82)	813 (80.18)			
	**Education**					2.780 (2)		.25
		College and below	289 (11.64)	61 (21.11)	228 (78.89)			
		Bachelor’s degree	1394 (56.14)	238 (17.07)	1156 (82.93)			
		Master’s degree and above	800 (32.22)	147 (18.38)	653 (81.63)			
	**Monthly income (¥)^a^**					1.569 (2)		.46
		≤5000	994 (40.03)	190 (19.11)	804 (80.89)			
		5001-10,000	884 (35.60)	150 (16.97)	734 (83.03)			
		>10,000	605 (24.37)	106 (17.52)	499 (82.48)			
**Positive psychological responses**								
	**Being confident**					80.584 (1)		<.001
		Yes	2339 (94.20)	380 (16.25)	1959 (83.75)			
		No	144 (5.80)	66 (45.83)	78 (54.17)			
	**Being hopeful**					47.537 (1)		<.001
		Yes	2359 (95.01)	395 (16.74)	1964 (83.26)			
		No	124 (4.99)	51 (41.13)	73 (58.87)			
	**Being rational**					113.526 (1)		<.001
		Yes	2281 (91.87)	354 (15.52)	1927 (84.48)			
		No	202 (8.13)	92 (45.54)	110 (54.46)			
**Information-seeking behaviors**								
	**Cannot stop searching information about the COVID-19 pandemic**					37.118 (1)	<.001	
		Yes	1474 (59.36)	322 (21.85)	1152 (78.15)			
		No	1009 (40.64)	124 (12.29)	885 (87.71)			
	**Concerned about the COVID-19 pandemic**					37.195 (1)	<.001	
		Yes	1223 (49.25)	278 (22.73)	945 (77.27)			
		No	1260 (50.75)	168 (13.33)	1092 (86.67)			
	**Time spent consuming information about the COVID-19 pandemic (hours)**					115.008 (2)	<.001	
		<1	906 (36.48)	92 (10.15)	814 (89.85)			
		1-2	1006 (40.52)	171 (17.00)	835 (83.00)			
		≥3	571 (23.00)	183 (32.05)	388 (67.95)			
	**Sources of information about the COVID-19 pandemic**							
	**Social media and commercial media**					9.826 (1)	.001	
		Yes	2159 (86.95)	408 (18.90)	1751 (81.10)			
		No	324 (13.05)	38 (11.73)	285 (87.96)			
	**Central official media**					3.004 (1)	.09	
		Yes	2104 (84.74)	366 (17.40)	1738 (82.60)			
		No	379 (15.26)	80 (21.11)	299 (78.89)			
	**Local official media, basic-level government and community**					0.901 (1)	.36	
		Yes	1508 (60.73)	262 (17.37)	1246 (82.63)			
		No	975 (39.27)	184 (18.87)	791 (81.13)			

^a^1¥=US $0.15

### Positive Psychological Responses

The prevalence of anxiety according to the participants' positive psychological responses are presented in Table 1. We observed that the prevalence rate of anxiety was lower for the participants who felt confident (2339/2483, 94.20%), hopeful (2358/2483, 95.01%), or rational (2281/2483, 91.87%) about the COVID-19 pandemic (*P*<.001).

### Information-Seeking Behaviors

The distribution and prevalence of anxiety with different information-seeking behaviors during the COVID-19 pandemic is shown in Table 1. In this study, participants who could not stop searching for information about the COVID-19 pandemic (1474/2483, 59.34%) or those who were concerned about the pandemic (1223/2483, 49.24%) were found to have a significantly higher prevalence of anxiety (*P<.*001) than other participants. The prevalence of anxiety was also observed to be higher for participants who spent more than 1 hour a day on searching for information about the COVID-19 pandemic (*P<.*001).

Our study findings also showed that participants who sought information about COVID-19 via social media and commercial media (2159/2483, 86.95%) had a higher prevalence of anxiety (408/2159, 18.90%) than those who did not seek information through social and commercial media (*P=.*001).

### Factors Associated With Anxiety

Table 2 shows the final results of the multivariable logistic regression analysis. Participants who were confident (odds ratio [OR] 0.434, 95% CI 0.243-0.775) or rational (OR 0.286, 95% CI 0.202-0.405) about the COVID-19 pandemic were found to be less likely to experience anxiety. In contrast, the information-seeking behaviors among the study participants, including cannot stop searching for information on COVID-19 (OR 1.593, 95% CI 1.236-2.052) and being concerned about the COVID-19 pandemic (OR 1.389, 95% CI 1.080-1.788) were found to be associated with anxiety. Moreover, participants spending more than 1 hour a day consuming information about the COVID-19 pandemic were also found to be more likely to report anxiety (1-2 h: OR 1.622, 95% CI 1.209-2.176; ≥3 h: OR 3.915, 95% CI 2.823-5.430). Additionally, participants who chose social media and commercial media as the primary source of information about the COVID-19 pandemic were observed to be more likely to report anxiety (OR 1.531, 95% CI 1.043-2.246).

**Figure 1:**
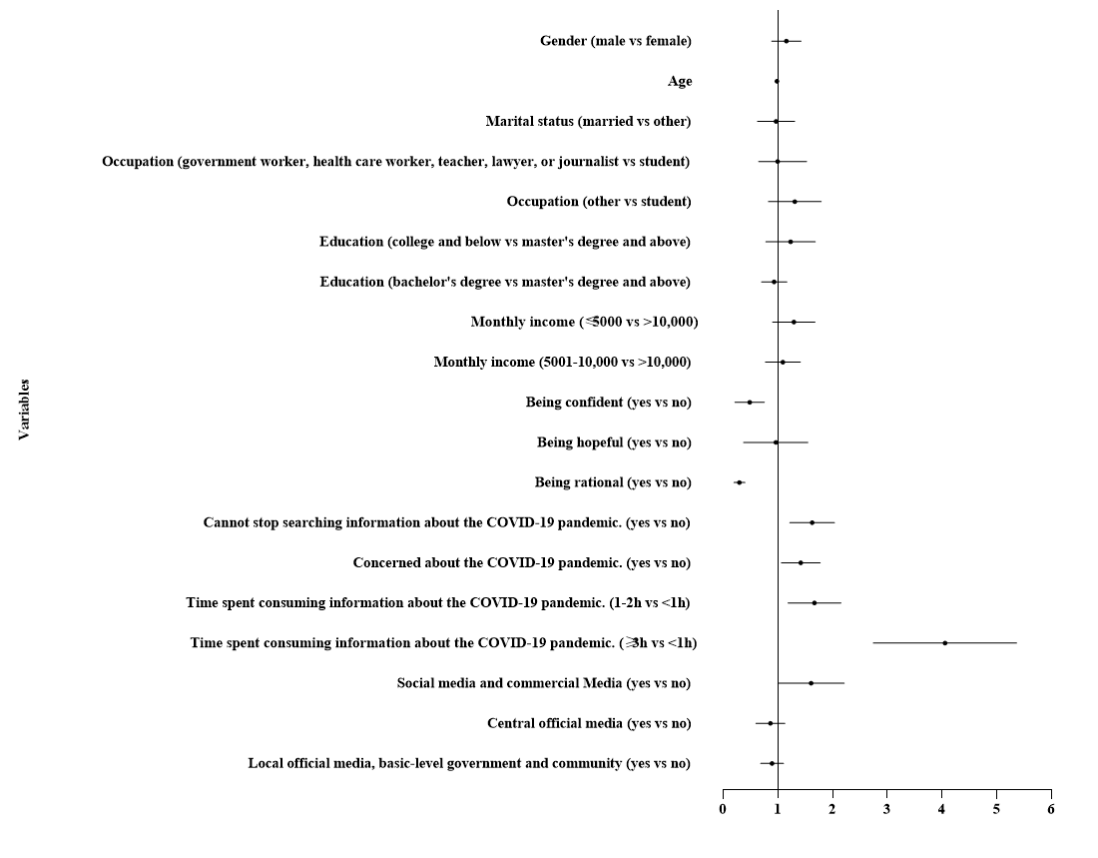
Forest plot of factors associated with anxiety.

Thus, the final model of multivariable logistic regression and forest plot (Figure 1) indicated that the following factors were associated with anxiety: cannot stop searching for information about the COVID-19 pandemic, being concerned about the COVID-19 pandemic, spending more than 1 hour a day consuming information about the COVID-19 pandemic, and use of social media and commercial media as the primary source of information about the COVID-19 pandemic. Conversely, being confident and being rational were independently found to be inversely associated with anxiety.

**Table 2:** Multivariable logistic regression analysis for factors associated with anxiety.

Variables			Odds ratio	95% CI
**Demographic characteristics**				
	Gender (male vs female)		1.135	0.898-1.435
	Age		0.981	0.967-0.995
	Marital status (married vs other)		0.927	0.648-1.327
	**Occupation**			
		Government worker, health care worker, teacher, lawyer, journalist (vs student)	0.995	0.649-1.527
		Other (vs student)	1.251	0.859-1.820
	**Education**			
		College and below (vs master’s degree and above)	1.177	0.810-1.711
		Bachelor’s degree (vs master’s degree and above)	0.913	0.710-1.174
	**Monthly income (¥)^a^**			
		≤5000 (vs >10,000)	1.253	0.925-1.699
		5001-10,000 (vs >10,000)	1.061	0.788-1.427
**Positive psychological responses**				
	Being confident (yes vs no)		0.434	0.243-0.775
	Being hopeful (yes vs no)		0.843	0.444-1.602
	Being rational (yes vs no)		0.286	0.202-0.405
**Information-seeking behaviors**				
	Cannot stop searching information on COVID-19 (yes vs no)		1.593	1.236-2.052
	Concerned about the COVID-19 pandemic (yes. vs no)		1.389	1.080-1.788
	**Time spent consuming information of COVID-19**			
		1-2 h (vs <1 h)	1.622	1.209-2.176
		≥3 h (vs <1 h)	3.915	2.823-5.430
	**Sources of information about the COVID-19 pandemic**			
		Social media and commercial media (yes vs no)	1.531	1.043-2.246
		Central official media (yes vs no)	0.836	0.611-1.143
		Local official media, basic-level government, and community (yes vs no)	0.877	0.693-1.111

^a^1¥=US $0.15

## Discussion

### Principal Findings

We observed that 17.96% of WeChat users in China who participated in this study reported having experienced anxiety during the early stages of the COVID-19 pandemic. This exceeds the prevalence of anxiety reported among the people in China before the pandemic (3.2%-8.1%) [52,53]. However, comparing the prevalence of anxiety during the severe acute respiratory syndrome (SARS) outbreak in 2002 (24.4%-35.0%), the prevalence of anxiety during the COVID-19 pandemic was slightly lower [54,55]. There remains a dearth of research on the relationship between information-seeking behaviors and anxiety during public health emergencies [56-58]. It is of vital importance to provide advice on how to cope with anxiety, including how to enhance positive psychological resources and prevent excessive information-seeking behaviors in order to prevent or reduce anxiety during public health emergencies. Our findings provide evidence-based recommendations on the promotion of positive psychological responses, psychological interventions, and information management to reduce anxiety for WeChat users in China.

Our study findings showed that anxiety was closely associated with positive psychological responses. Among the positive psychological responses, being confident and being rational were observed to be associated with lower prevalence of anxiety, which suggests that being confident and rational might prompt individuals to cope better with adverse events and difficult challenges during public health emergencies. Being rational also has been found to influence anxiety in previous studies, wherein people who were more positive coped better with life stressors and had low levels of anxiety [59,60]. WeChat users in this study had been exposed to a variety of stressors such as the perception of severe health risks of COVID-19, negative emotions associated with home quarantine, financial hardships caused by delays in work, and uncertainty during the pandemic, which might have resulted in reduced positive psychological responses and higher prevalence of anxiety. The framework of intolerance of uncertainty states that intolerance of uncertainty and cognitive avoidance are positively correlated with psychological responses and anxiety [23,61,62].

People could cope with stressors better and improve their mental health by means of rational-emotive behavioral interventions and cognitive behavioral therapies to reduce anxiety and improve psychological abilities, including emotion regulation and stress management [28,29,63,64]. The emotion dysregulation model of anxiety suggests that the lack of emotion regulation has a positive correlation with anxiety [65,66]. Therefore, to intensify protective effects of positive psychological responses, psychological interventions for the public might be an effective approach, and these should be provided as soon as possible during public health emergencies.

This study also found that excessive consumption of information about COVID-19 might be closely linked with anxiety. Consuming information about the pandemic for more than 1 hour a day was observed to be associated with anxiety. Our results are consistent with other studies showing that anxiety is associated with increased use of smart phones, leading to excessive exposure to COVID-19 news [19,22,67,68]. The frequency and duration of searching for health information could exacerbate stress, anxiety, and perception of health risks [69-72]. WeChat users in this study might actively search or passively receive health information about the COVID-19 pandemic via multiple media sources. Searching for health information can be regarded as a source of anxiety and negative psychological responses. Spending too much time searching for health information could be linked to increased levels of anxiety, anger, or sadness. This might be due to increased fear of being infected by a severe disease and could adversely impact people’s self-esteem and reduce their tolerance of uncertainty [73-81]. Conversely, it is also possible that anxious people are more likely to overconsume information. Health professionals should provide timely suggestions for the public on how to access and absorb health information from trusted sources [77,82,83].

Moreover, sources of information about COVID-19 were found to be significantly associated with anxiety, especially information obtained via social media was observed to have a positive correlation with anxiety. Excessively searching for health information on social media during traumatic events (eg, public health emergencies, food safety incidents, terror attacks, and natural disasters) can result in sleep disorders, distress such as anxiety and depression, and posttraumatic stress disorder [84-89]. Social media provides open platforms for the public to exchange their ideas and perspectives in a timely and prompt manner. However, it might be difficult for the public to distinguish true information from false information. Through improved dissemination of health information, coping strategies and healthy behaviors in the context of the COVID-19 pandemic could be encouraged, in addition to focusing on the prevention of negative psychological responses and improvement on positive psychological responses [90].

### Limitations

Several limitations should be acknowledged. First, the data were collected using a self-administered web-based questionnaire via smartphones, which may have resulted in information bias and misclassification bias. It is possible that participants did not provide accurate information in the study in order to either be included in the study or to move quickly through the survey. To minimize the bias as much as possible, a pilot study was conducted to acquire participants’ perception of the questionnaire. Therefore, the collected questionnaire had been filtered with data cleaning, checking for consistency and logicality of the answers, adjusting invalid and missing values. Due to the severity of the COVID-19 pandemic and the necessity of timely acquisition of data, the survey was distributed via WeChat rather than conducted using a face-to-face approach. Additionally, this survey among WeChat users used a convenience sampling method during the first 2 months of the COVID-19 pandemic. Therefore, generalizability of the results may be limited to the whole population of China. As the survey was shared within academic WeChat groups, it is possible the results reported in our study are from a population that has a higher education level than the general population, and this may have affected anxiety levels either positively or negatively, because the participants likely had a stronger understanding of the pandemic. Future studies should be carried out with random sampling. Finally, the results may be limited by residual confounding from unmeasured confounders such as regional economic disparity, allocation of health resources, and an individual’s social status. To minimize the potential errors induced by unmeasured confounders, the survey was conducted across 3 municipalities and 22 provinces via WeChat, one of the most popular social platforms in China.

### Conclusions

The COVID-19 pandemic has adversely affected the mental health of the public and resulted in a high prevalence of anxiety among them, especially during the early stages of the pandemic. Certain behaviors such as cannot stop searching for information on COVID-19, being concerned about the COVID-19 pandemic, spending more than 1 hour a day consuming information about the COVID-19 pandemic, and using social media and commercial media as the primary source of information about the COVID-19 pandemic were found to be associated with anxiety. In contrast, positive psychological responses such as being confident and being rational were found to be negatively correlated with anxiety. Prompt measures and guidance from public health authorities on choosing reliable and trusted sources of information might decrease the negative effects of overconsumption of COVID-19–related information and improve the overall mental health of the public.

## Appendix

Multimedia Appendix 1 The multivariable logistic regression analysis of anxiety (without positive psychological responses).

## Abbreviations

GAD-7: Generalized Anxiety Disorder 7-item
KMO: Kaiser-Meyer-Olkin
OR: odds ratio
SARS: severe acute respiratory syndrome
