# Supplementary material for: Factors Influencing Anxiety Among WeChat Users During the Early Stages of the COVID-19 Pandemic in Mainland China: Cross-sectional Survey Study
Source: J Med Internet Res. 2021 May 17;23(5):e24412. doi: 10.2196/24412 (PMC8130820; doi:10.2196/24412)
Supplement: Multimedia Appendix 1 [file jmir_v23i5e24412_app1.doc]

**Multimedia Appendix 1. The multivariable logistic regression analysis of anxiety (without positive psychological responses)**

| Variables | OR | 95%CI |
| --- | --- | --- |
| *Demographic characteristics* |  |  |
| Gender (male vs female) | 1.125 | 0.896-1.413 |
| Age (years) | 0.980 | 0.967-0.994 |
| Marital status (married vs other) | 0.876 | 0.618-1.242 |
| Occupation |  |  |
| Government worker, health care worker, teacher, lawyer, journalist vs student | 1.028 | 0.679-1.557 |
| Other vs student | 1.399 | 0.976-2.005 |
| Education |  |  |
| College and below vs master and above | 1.152 | 0.802-1.654 |
| Bachelor’s degree vs master’s degree and above | 0.925 | 0.725-1.180 |
| Monthly income (¥^a^) |  |  |
| ≤5000 vs>10,000 | 1.267 | 0.942-1.704 |
| 5001 to 10,000 vs>10,000 | 1.035 | 0.776-1.382 |
| *Information seeking behaviors* |  |  |
| Cannot stop searching information on COVID-19 (yes vs no) | 1.562 | 1.222-1.998 |
| Concern about the COVID-19 pandemic. (yes vs no) | 1.304 | 1.023-1.663 |
| Time spent consuming information of COVID-19 |  |  |
| 1-2h vs <1h | 1.682 | 1.264-2.238 |
| ≥3h vs <1h | 4.075 | 2.969-5.594 |
| Sources of information about the COVID-19 pandemic. |  |  |
| Social media and commercial media (yes vs no) | 1.536 | 1.058-2.231 |
| Central official media (yes vs no) | 0.777 | 0.575-1.050 |
| Local official media, basic-level government and community (yes vs no) | 0.841 | 0.669-1.058 |

^a^1 ¥ = US $0.15”
